# Tooth Loss Is Associated With Increased Risk of Dementia and With a Dose-Response Relationship

**DOI:** 10.3389/fnagi.2018.00415

**Published:** 2018-12-18

**Authors:** Jun Chen, Chang-Ju Ren, Lan Wu, Ling-Yun Xia, Jun Shao, Wei-Dong Leng, Xian-Tao Zeng

**Affiliations:** ^1^Department of Neurology, Taihe Hospital, Hubei University of Medicine, Shiyan, China; ^2^Department of Evidence-Based Medicine and Clinical Epidemiology, Center for Evidence-Based and Translational Medicine, Zhongnan Hospital of Wuhan University, Second Clinical College of Wuhan University, Wuhan, China; ^3^Department of Stomatology, Taihe Hospital, Hubei University of Medicine, Shiyan, China; ^4^Department of Stomatology, Guangzhou Hospital of Integrated Traditional and West Medicine, Guangzhou, China

**Keywords:** tooth loss, periodontal disease, dementia, risk factor, dose-response analysis, meta-analysis

## Abstract

**Objective:** Both tooth loss and dementia are age-related and frequently-occurring diseases. Increasing attention has been given to explore the pathogenesis related to oral-brain function disorders. The present study was performed to evaluate the association between tooth loss and dementia through a dose-response meta-analysis.

**Methods:** Relevant cohort studies were searched from online databases up until June 20, 2018, which examined the association between tooth loss and the risk of dementia. Literature selection according to inclusion and exclusion criteria, as well as data extraction from included studies were completed independently by two reviewers. Data syntheses in this meta-analysis were performed using Stata 12.0 software.

**Results:** A total of 8 cohort studies were included, containing a total of 14,362 samples and 2,072 dementia patients. The result of the meta-analysis indicated that patients with tooth loss faced a 1.34 times greater risk of developing dementia (RR = 1.34,95% CI = 1.19-1.51). The result from this dose-response meta-analysis in a linear model, suggested that every missed tooth might increase the risk of dementia by 1.01 times (RR = 1.01, 95%CI = 1.00-1.02). Further subgroup analyses pointed out that tooth loss patients without dentures may have a higher risk of dementia than those with dentures (with denture: RR = 0.98, 95% CI = 0.87-1.10; without denture: RR = 1.53, 95% CI = 1.19-1.97); at the same time, the study design, study area and education level of the study participants, might also have some effect on the results.

**Conclusions:** Tooth loss may be a risk factor for the development of dementia. In addition, there is a dose-response relationship with the increase of missing teeth.

## Introduction

Tooth loss frequently occurs in patients with dental caries, periodontal diseases, tumors, traumatism and retrogression, accompanied by masticatory dysfunction, hypotrophic absorption, pronunciation difficulty and anile appearance. Nowadays, tooth loss is no longer regarded as a simple local disorder, but also as a risk factor for systemic diseases, such as cardiovascular diseases (Cheng et al., 2018), cancers (Zeng et al., 2013; Shi et al., 2018), obesity (Nascimento et al., 2016), stroke (Lafon et al., 2014), and mental illnesses (Kisely et al., 2015).

Dementia is a disease with progressive degeneration of the central nervous system, with Alzheimer's disease (AD) as the most common type and accounts for 60–70% of all dementia cases (Mayeux, 2003). The main clinical features of dementia include cognitive impairment, abnormal mind and behavior and decreased ability of daily life. To our knowledge, both tooth loss and dementia are common age-related diseases (Busby et al., 2014; Lawton et al., 2015), and along with the progression of population aging, increasing attention has been devoted to the association between oral-brain function disorders (Lehrer et al., 2015; Kothari et al., 2016; Pillai et al., 2018), tooth loss and dementia (Chen et al., 2010; Minn et al., 2013; Singhrao et al., 2014). Recently, several systematic reviews and meta-analyses have examined the link between tooth loss and cognitive status (Shen et al., 2016; Tonsekar et al., 2017; Oh et al., 2018). Based on existing studies, we conducted a dose-response analysis on relevant cohort studies to provide updated evidence on the relationship between the number of teeth lost and dementia.

## Materials and Methods

Our study conformed to the recommended Preferred Reporting Items for Systematic Reviews and Meta-Analyses (PRISMA) statement (Moher et al., 2009).

### Inclusion and Exclusion Criteria

According to the instruction of the “PICOS (population, exposure, control, outcome, and study design),” eligible studies were selected based on the following inclusion criteria: (1) interested population consisted of patients suffering tooth loss and/or dementia without restrictions on gender, age and race; (2) interested exposure was tooth loss; (3) participants in the control group had no tooth loss; (4) interested outcome was referred to dementia; (5) study design restrictively followed cohort design; and (6) with full text and relevant data as follows: hazard ratio (HR), odds ratio (OR), relative risk (RR) with 95% confidential interval (CI) or raw data for calculating crude HR, OR, or RR with corresponding 95% CI. If outcomes were duplicated or shared in more than one study from one institution, the most comprehensive study was chosen for the analysis.

Studies would be excluded if they conformed to any one of the following conditions: (1) animal experiments; (2) focusing on risk factors (such as periodontitis and caries) for tooth loss but without risk estimation on tooth loss; (3) interested outcomes could only be classified into clinical manifestations of dementia (such as cognitive decline, memory impairment and daily living ability decline), but did not conform to dementia itself.

### Search Strategy

We searched the databases of PubMed, Embase, China National Knowledge Infrastructure (CNKI) and the International Clinical Trial Registry Platform (ICTRP) for relevant cohort studies published before June 20, 2018, and the references of relevant papers were also examined to detect additional studies. The following items were applied in literature searching: “tooth loss” or “edentulous” or “edentulism” or “periodontal disease” combined with “dementia” or “Alzheimer's disease” or “risk factor.”

### Study Selection and Data Extraction

The studies finally included were selected according to inclusion and exclusion criteria. A designed form was then utilized to collect the following information from the included studies: the name of the first author, year of publication, the type of study design, study area, sample size, participants' age, follow-up duration, cognitive assessment, tooth loss assessment, risk estimation results (OR, RR, HR with the corresponding 95%CI), and adjustment for covariates. Both the study selection and data extraction were conducted by two reviewers independently, cross checked and any disagreements over the data between the reviewers, were resolved through discussion.

### Statistical Analysis

We assessed the association between tooth loss and dementia, through pooling RRs and corresponding 95% CIs, for the highest risk vs. the lowest risk. HRs were directly considered to be equivalent to RRs, and ORs were also treated equally as a low prevalence of dementia in all populations. Additionally, a subgroup analysis was conducted to clarify the impact of pooled HRs and ORs on the overall result. Heterogeneity was investigated using *I*^2^ statistics and a *Q* test, and the fixed-effect model was utilized when *I*^2^ ≤ 50% and *p* > 0.1; otherwise the random-effect model was preferred (Melsen et al., 2014). Further subgroup analyses were performed based on the study area, study design, follow-up duration, denture status and some adjustment factors, respectively. In addition, a dose-response analysis was also conducted to explore the relationship between tooth loss and dementia based on a multi-category analyses according to the number of missing teeth (Greenland and Longnecker, 1992), and a data conversion was performed when the number of remaining teeth was assessed. Moreover, publication bias among included studies was examined through funnel plots and an Egger's test, and a symmetrical funnel plot where *P* > 0.05 indicated no significant publication bias (Egger et al., 1997; Song and Gilbody, 1998). All of the statistical analyses mentioned above were accomplished using Stata 12.0 software (Orsini et al., 2012).

## Results

### Study Selection and Characteristics

**Figure 1:**
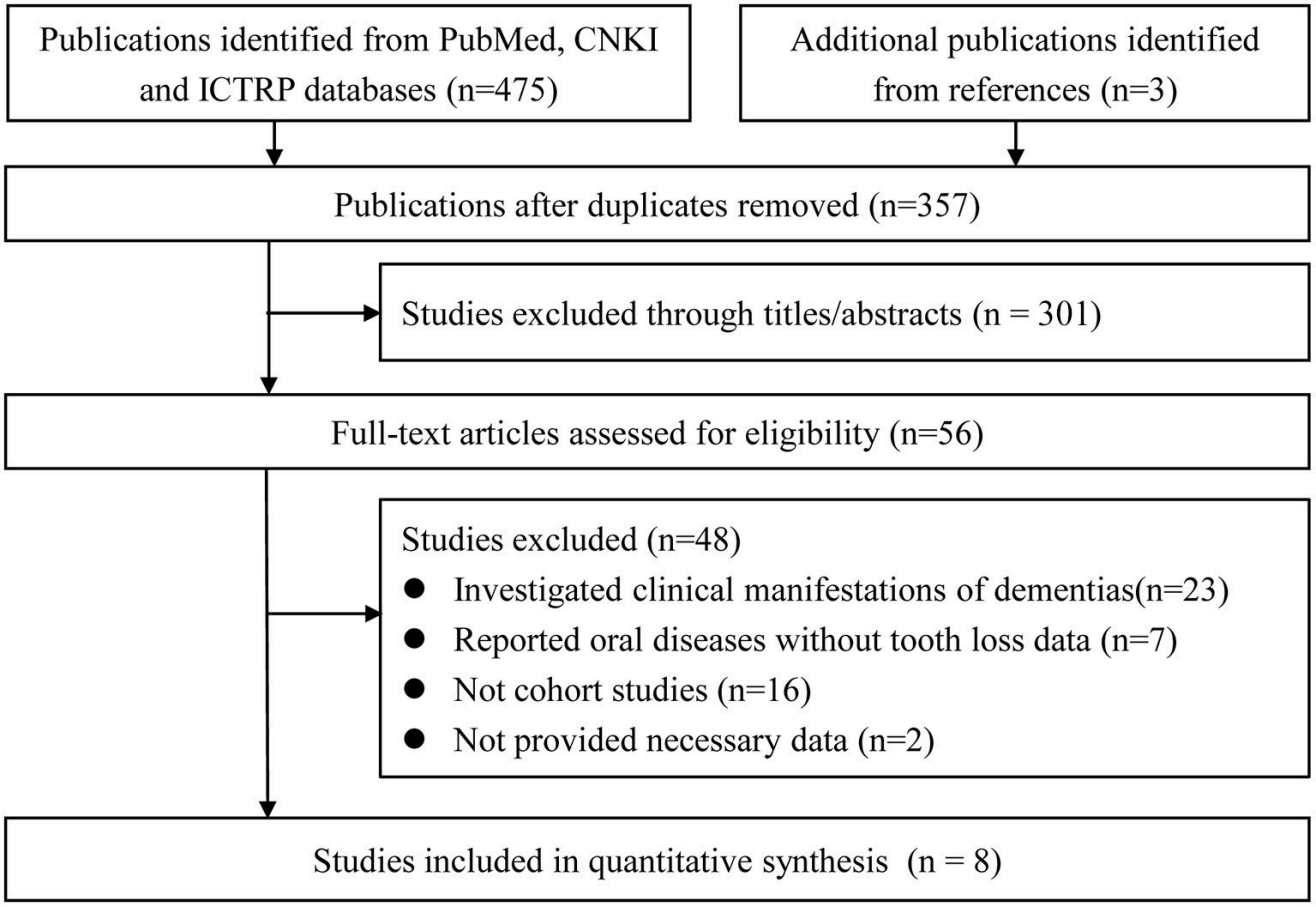
Flow diagram for the study selection.

A total of 478 potentially relevant studies were firstly retrieved. Of those, 357 remained after eliminating duplicates, while 56 entered a full-text examination after assessing the titles and abstracts. Finally, eight studies (Shimazaki et al., 2001; Stein et al., 2007; Arrive et al., 2012; Paganini-Hill et al., 2012; Yamamoto et al., 2012; Batty et al., 2013; Stewart et al., 2015; Takeuchi et al., 2017) were included in the meta-analysis. The detailed process is shown in Figure 1.

The eight eligible cohort studies were published in English, between 2001 and 2017, and included five prospective (Shimazaki et al., 2001; Arrive et al., 2012; Yamamoto et al., 2012; Batty et al., 2013; Takeuchi et al., 2017) and three retrospective (Stein et al., 2007; Paganini-Hill et al., 2012; Stewart et al., 2015) cohort studies, containing a total of 14,362 samples and 2,072 dementia patients (Shimazaki et al., 2001; Stein et al., 2007; Arrive et al., 2012; Paganini-Hill et al., 2012; Yamamoto et al., 2012; Batty et al., 2013; Stewart et al., 2015; Takeuchi et al., 2017). Among these studies, three reported discrete results for some variables, including denture status (with or without dentures; Shimazaki et al., 2001), educational level (higher or lower level; Arrive et al., 2012), and gender (male or female; Paganini-Hill et al., 2012). Consequently, each of these three reports offered two independent studies for analyses, so the final meta-analyses contained 11 datasets. Of the included publications, eight data sets obtained from six studies provided more than two classifications according to the number of teeth (Shimazaki et al., 2001; Stein et al., 2007; Paganini-Hill et al., 2012; Batty et al., 2013; Stewart et al., 2015; Takeuchi et al., 2017). And most of the studies included, reported the results using HRs; while the others used ORs. Most of the reports chose the number of remaining teeth, for tooth loss assessment, but one report (Arrive et al., 2012) adopted the number of missing teeth for the assessment. Other influential differences also existed across the studies included, such as study area, follow-up duration, denture status and important risk factors. More detailed characteristics of these studies are shown in Tables 1, 2.

**Table 1:** Characteristics of included studies.

**Study**	**Regions; Name of cohort**	**Study design**	**Age(years)**	**Follow-up times (years)**	**Sample size**	**Cognitive assessment**	**Tooth loss assessment**	**Results**
Shimazaki et al., 2001	Japan; Kitakyushu City	Prospective cohort	59–107	6	156/517	Medical records; Incident and mortality dementia	Number of remaining teeth (reference ≥20 teeth)	With denture, OR (95%CI) 1–19 teeth: OR 1.9 (0.8–4.6) Edentulous:1.7 (0.7–4.0) Without denture, OR (95%CI) 1–19 teeth: 2.3 (0.9–5.8) Edentulous: 2.4 (0.9–6.5)
Stein et al., 2007	USA; the Nun Study	Retrospective cohort	40–75	12	32/144	the Mini-Mental State Examination and Activities of Daily Living; Incident dementia	Number of remaining teeth excluding non-third molars (reference 10–28 teeth)	HR (95%CI): 17–28: Reference 10–16:HR 0.4 (0.10–1.76) 1–9:HR 1.8 (0.58–5.46) 0: HR 0.9 (0.25–3.12) 10–28: Reference 0–9 teeth: HR 2.20(1.1–4.5)
Arrive et al., 2012	France; The Personnes Age'es QUID (PAQUID) Study	Prospective cohort	66–80	15	72/405	DSM-III Revised; Incident dementia	Number of missing teeth (reference < 11 teeth)	Higher school level, HR (95%CI): ≥11 teeth: 1.07(0.57–2.02) Unified: 0.93(0.5–1.75) ^#^ Lower school level: ≥11 teeth: 0.30(0.11–0.79) Unified: 3.33(1.27–8.73) ^#^
Paganini-Hill et al., 2012	USA; The Leisure World Cohort Study	Retrospective cohort	52–105 (median 81)	18	1145/5468	Follow-up questionnaires, hospital records, death certificates, and in-person evaluations; Incident dementia	Number of remaining teeth (reference ≥26 teeth)	Men, HR (95%CI): 16–25 teeth: 1.17 (0.86–1.58) 1–15 teeth: 1.21 (0.84–1.73) 0 teeth: 1.20 (0.77–1.87) Women, HR (95%CI) 16–25 teeth: 0.94 (0.80–1.10) 1–15 teeth: 0.96 (0.79–1.17) 0 teeth: 1.07 (0.84–1.36)
Yamamoto et al., 2012	Japan; the Aichi Gerontological Evaluation Study (AGES) Project	Prospective cohort	≥ 65	4	220/4425	Standardized questionnaire; Incident dementia	Number of remaining teeth (reference ≥20 teeth)	HR (95%CI) with/without dentures: ≤ 19 teeth: 1.01(0.67–1.51) Few teeth with dentures: 2.70 (1.84–3.94) Few teeth without dentures 4.57 (2.63–7.94)
Batty et al., 2013	UK; Action in Diabetes and Vascular disease: Preterax and Diamicron Modified-Release Controlled Evaluation (ADVANCE) trial	Prospective cohort	55–88	5	109/1140	DSM-IV	Number of remaining teeth (reference ≥22 teeth)	HR (95%CI): 1–21 teeth: 1.24 (1.05, 1.46) 0 teeth: 1.48 (1.24, 1.78)
Stewart et al., 2015	Sweden; the Prospective Population Study of Women (PPSW) in Gothenburg	Retrospective cohort	25–74	37	158/697	DSM-III Revised; Incident dementia	Number of remaining Teeth(reference ≥25 teeth)	Women, OR (95%CI) ≥25 teeth: 21–24 teeth:1.24 (0.74–2.09) 9–20 teeth: 1.25 (0.72–2.17) <9 teeth: 1.81 (1.03–3.19) Lowest vs. Highest Quartile: 1.62 (0.84–3.11)
Takeuchi et al., 2017	Japan; The Hisayama Study	Prospective cohort	≥60	5	180/1566	DSM-III Revised; Incident dementia	Number of remaining Teeth(reference ≥20 teeth)	HR (95% CI) 10–19 teeth:1.62 (1.06–2.46) 1–9teeth: HR 1.81 (1.11–2.94) 0 teeth: HR 1.63 (0.95–2.80)

*DSM-IV, Diagnostic and Statistical Manual of Mental Disorders-IV; HR, hazard ratio; OR, odds ratio; CI, confidential interval; CCD, Cardiovascular and cerebrovascular diseases*.

# *Unified data were from the reciprocal of the original data*.

**Table 2:** Adjustment status of included studies.

**Study**	**Adjustment for covariates**	**Adjustment strength**
Shimazaki et al., 2001	Age, physical health, type of institution, cerebrovascular disorder, denture use	Weak
Stein et al., 2007	Age, education and apolipoprotein E4 allele	Weak
Arrive et al., 2012	Gender, body mass index, diabetes, depression, hypertension and ischemic cardiopathy / history of brain stroke	Weak
Paganini-Hill et al., 2012	Age at entry, smoking, alcohol, caffeine, active activities, other activities, body mass index, high blood pressure, angina pectoris, heart attack, stroke, diabetes mellitus, rheumatoid arthritis, cancer, education, head trauma, and family history of dementia	Strong
Yamamoto et al., 2012	Age, household income, body mass index, present illness, alcohol consumption, exercise, and forgetfulness	Weak
Batty et al., 2013	Age, sex, European Quality of Life-5 Dimensions (EQ-5D), socioeconomic, cigarette smoking, alcohol intake, vigorous physical activity in previous week, Hemoglobin A1c, creatinine, body mass index, total cholesterol, HDL cholesterol, resting heart rate, systolic blood pressure, diastolic blood pressure, age at completion of highest level of education	Strong
Stewart et al., 2015	Age, education, social class, stroke, myocardial infarction, diabetes mellitus, smoking status, systolic blood pressure, body mass index, and cholesterol level defined in quartile groups with pacifier categories for missing data	Strong
Takeuchi et al., 2017	Sex, age, occupation, education, hypertension, diabetes mellitus, history of stroke, alcohol intake, tooth brushing frequency, regular visits to the dentist, and denture use	Strong

### Overall Analysis

**Figure 2:**
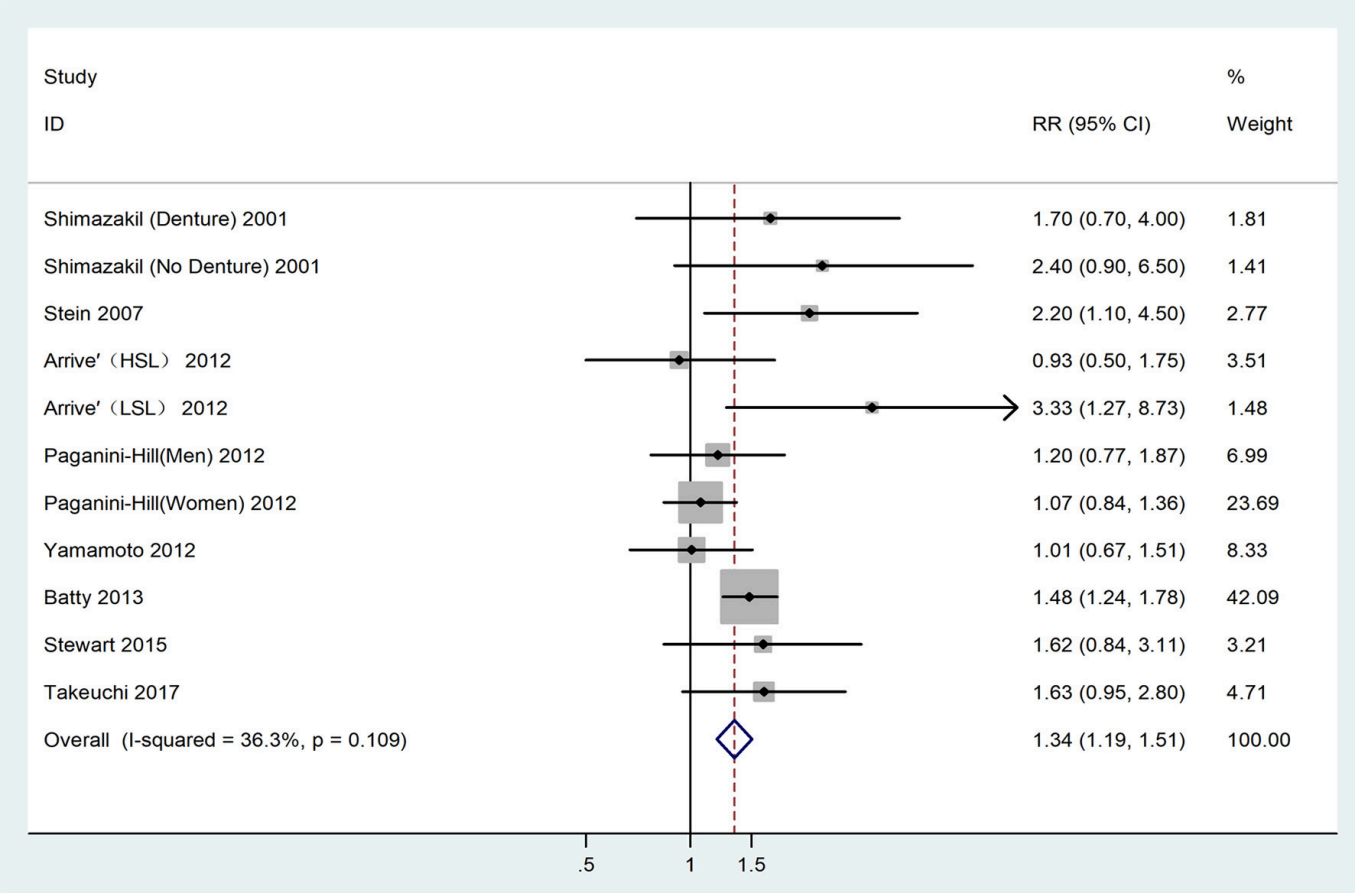
Forest plot for the connection between tooth loss and dementia risk among the overall population. HSL, Higher school level; LSL, Lower school level.

The heterogeneity detected between the 11 datasets, was low (*I*^2^ = 36.30%, *P* = 0.11), and determined the adoption of the fixed-effect model for analysis. The analysis result indicated that patients with tooth loss had a 1.34 times higher risk of developing dementia (RR = 1.34,95% CI = 1.19-1.51), as shown in Figure 2.

### Dose-Response Analysis

**Figure 3:**
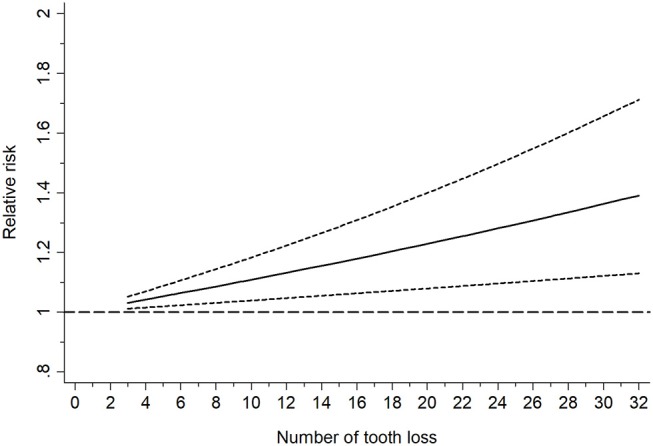
Linearly based dose-response analysis on the relationship between tooth loss and dementia risk.

A further analysis investigated the dose-response relationship of the two diseases based on six studies with eight datasets (Shimazaki et al., 2001; Stein et al., 2007; Paganini-Hill et al., 2012; Batty et al., 2013; Stewart et al., 2015; Takeuchi et al., 2017), as shown in Figure 3. No non-linear relationship was detected (*P* = 0.52) and the dose-response meta-analysis suggested that every missing tooth might increase the risk of dementia by 1.01 times (RR = 1.01, 95%CI = 1.00-1.02) in a linear model, with the risk increasing to 1.18 times, when the number of missing teeth went up to 20 (RR = 1.18, 95%CI = 1.10-1.27). Correspondingly, edentulous cases faced a 1.31 times higher risk of dementia (RR = 1.31, 95%CI = 1.17-1.47).

### Subgroup Analyses

The subgroup analyses further discussed some influential factors and assessed the stability of the results according to the type of study design, study area, follow-up duration, denture status, risk expression, and adjustments for confounding factors, as shown in Table 3. No significant differences were found in the subgroup analyses, based on of follow-up duration, risk expression, the strength of adjustment and adjustments for smoking, alcohol, and cardiovascular and cerebrovascular diseases; while significant differences were detected when stratified by the study design, study area, denture status, and adjustment for education. The study design was dramatically related to the final result (Prospective cohort: RR = 1.17, 95% CI = 0.97-1.41; Retrospective cohort: RR = 1.24, 95% CI = 1.12-1.37). When stratified by the study area, a significant association was only found for those conducted in Europe (Asia: RR = 1.33, 95% CI = 0.99-1.77; America: RR = 1.16 95% CI = 0.95-1.42; Europe: RR = 1.48, 95% CI = 1.25-1.74). Eight data sets obtained from three publications were used to analyze the risk of dementia related to tooth loss among the patients with and without dentures (Shimazaki et al., 2001; Paganini-Hill et al., 2012; Yamamoto et al., 2012) and the results showed a distinct relationship in the no-denture group only (with denture: RR = 0.98, 95% CI = 0.87-1.10; without denture: RR = 1.53, 95% CI = 1.19-1.97). A significant association was detected for the study's results, with an adjustment for education, while the significance disappeared when lacking such adjustments (Yes: RR = 1.36, 95% CI = 1.19-1.54; No: RR = 1.45, 95% CI = 0.92-2.29).

**Table 3:** Results of the meta-analysis.

**Overall and subgroup analysis**	**No. of studies**	**Meta-analysis**		**Effect model**	**Heterogeneity test**		
		**RR (95%CI)**	***P*-value**		***I^**2**^*(%)**	***P*-value within group**	***P*-value between group**
Overall	11	1.34(1.19–1.51)	0.00	Fixed	36.3	0.11	–
**STUDY DESIGN**							
Prospective cohort	7	1.43(1.23–1.66)	0.00	Fixed	34.4	0.17	0.16
Retrospective cohort	4	1.20(0.99–1.45)	0.07	Fixed	33.7	0.21	–
**STUDY AREA**							
Asia	4	1.33(0.99–1.77)	0.06	Fixed	24.6	0.26	0.20
America	3	1.16(0.95–1.42)	0.14	Fixed	44.8	0.16	–
Europe	4	1.48(1.25–1.74)	0.00	Fixed	36.3	0.18	–
**FOLLOW-UP TIME**							
<10	5	1.44(1.23–1.55)	0.00	Fixed	8.7	0.36	0.17
≥10	6	1.36(1.01–1.83)	0.046	Random	47.1	0.09	–
**DENTURE STATUS**							
With denture	4	0.98(0.87–1.10)	0.74	Fixed	<0.01	0.93	<0.01
Without denture	4	1.53(1.19–1.97)	0.00	Fixed	14.7	0.32	–
**RISK EXPRESSION**							
HR	4	1.27(1.04–1.46)	0.02	Random	47.9	0.08	0.06
OR	7	1.90(1.29–2. 80)	0.00	Fixed	<0.01	0.88	–
**ADJUSTMENT STRENGTH**							
Weak	6	1.56(1.03–2.36)	0.04	Random	50.5	0.07	0.88
Strong	5	1.33(1.17–1.53)	0.00	Fixed	28.2	0.23	–
**ADJUSTMENT FOR SMOKING**							
Yes	4	1.32(1.19–1.51)	0.00	Fixed	40.1	0.17	0.62
No	7	1.41(1.11–1.79)	0.01	Fixed	42.5	1.11	–
**ADJUSTMENT FOR ALCOHOL**							
Yes	5	1.29(1.14–1.46)	0.00	Fixed	40.7	0.15	0.11
No	6	1.69(1.24–2.41)	0.00	Fixed	22.3	0.27	–
**ADJUSTMENT FOR EDUCATION**							
Yes	6	1.36(1.19–1.54)	0.00	Fixed	32.9	0.19	0.64
No	5	1.45(0.92–2.29)	0.11	Random	50.2	0.09	–
**ADJUSTMENT FOR CCD**							
Yes	8	1.25(1.05–1.48)	0.01	Fixed	31.0	0.18	0.28
No	3	1.40(1.00–1.63)	0.047	Random	54.4	0.11	–

*RR, relative risk; CI, confidence interval; HR, hazard ratio; OR, odds ratio; CCD, Cardiovascular and cerebrovascular diseases*.

### Publication Bias

**Figure 4:**
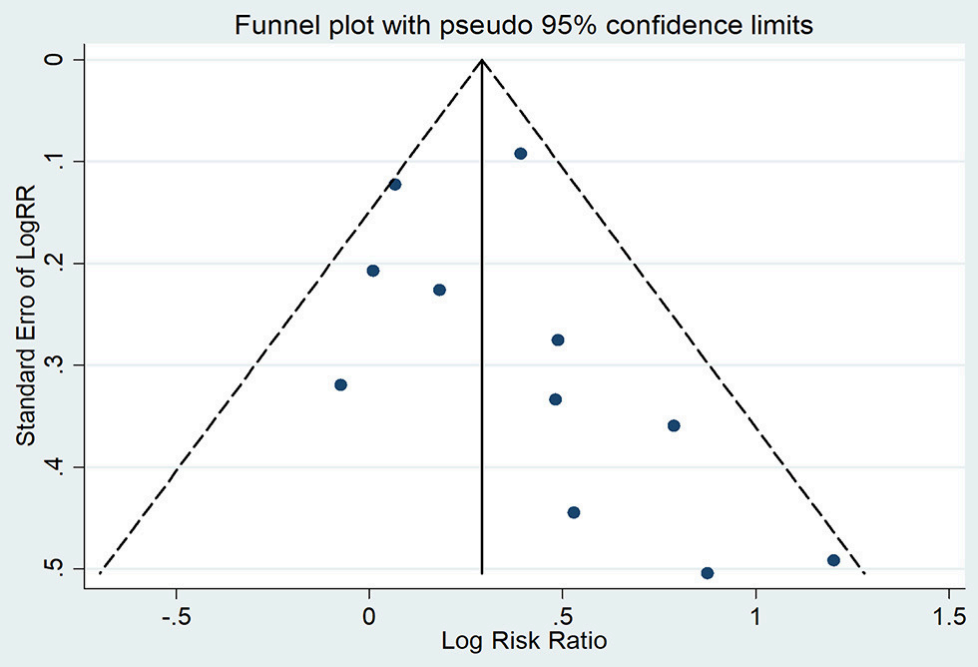
Funnel plot for publication bias among overall population.

The symmetry in Figure 4 is not perfect, but still implied the absence of significant publication bias, which was confirmed by Egger's test (*P* = 0.29).

## Discussion

Our present study investigated the association between tooth loss and dementia based on eight cohort studies involving a total of 14,362 cases and 2,072 dementia patients. The result of this meta-analysis indicated that tooth loss statistically increased the risk of dementia by 1.34-fold. A further dose-response analysis detected a linearity association between the two diseases, suggesting that the more teeth are missing, the higher the risk of dementia. In subgroup analyses, we found that the association between the two illnesses might be affected by certain factors, such as study design, study area, denture status and education level, but was not affected by follow-up duration, risk expression, the strength of confounding factor adjustments, or adjustments for smoking, alcohol and cardiovascular and cerebrovascular diseases.

A positive association between the two diseases, detected in tooth loss patients without dentures, might be partially explained by the alteration of chewing ability (Savikko et al., 2013; Teixeira et al., 2014). A further explanation may include that masticatory dysfunction after tooth loss, may lead to decreased cerebral blood circulation and neurotransmitter secretion (Lin, 2018). Some scholars think that masticatory movement could promote blood supply to the brain and then prevent dementia, based on the fact that prosthodontic treatment with dentures can effectively improve the disorders described above (Yamamoto et al., 2012; Luraschi et al., 2013; Banu et al., 2016). Periodontal disease, a frequently discussed cause of tooth loss, can stimulate the release of some cytokines which are related to the activation of molecules contributing to neurodegeneration, such as C-reactive protein (CRP), tumor necrosis factor-α (TNF-α), immunoglobulin G (IgG), interleukin-1β (IL-1β), and interleukin-6 (IL-6) (Kamer et al., 2009; Koyama et al., 2013; Dursun et al., 2015; Liesz et al., 2015). Nutritional deficiency related to tooth loss has also been regarded as a marker for dementia, especially when the deficiency involves lessened intakes of protein, essential vitamins such as sodium, vitamin D, vitamin B1, vitamin B6, niacin, pantothenic acid, phospholipids, and other micronutrients, all of which are essential for the formation of new synapses associated with human memory (Perera and Ekanayake, 2012; Engelborghs et al., 2014). Some researchers have also suggested that essential nutrients can not only reduce the risk of oral diseases, including tooth loss, but can also reduce the susceptibility to dementia (Kim et al., 2007; Jimenez et al., 2014). Possible mechanisms of such a decline may be related to certain functions of some essential nutrients. For example, vitamin D has been reported to be able to inhibit the activity of some cytokines related to periodontitis and dementia by decreasing IL-6, IL-8, and TNF-α expression (Tang et al., 2013; Jayedi et al., 2018).

Apart from the present meta-analysis, three previously published meta-analyses have explored the association between tooth loss/ periodontitis and dementia, and revealed that tooth loss might be a risk factor for the development of dementia (Shen et al., 2016; Tonsekar et al., 2017; Oh et al., 2018). Additionally, these three meta-analyses not only paid close attention to dementia, but also to cognitive decline, such as the studies by Oh et al. (2018) and by Tonsekar et al. (2017) based on cohort studies; and Shen et al. (2016) based on cross-sectional, case-control, and cohort studies. Based on published articles, our study targeted reports with confirmed diagnosis criteria for dementia and cohort design. In addition, we adopted dose-response analysis to discuss the correction of the tooth number and the level of dementia risk, and subgroup analyses clarified possible confounding factors affecting the result. What's more, the results were reliable considering the homogeneity between the studies that were included (*I*^2^ = 36.30%, *P* = 0.11) and the lack of significant publication bias (*P* = 0.29).

However, there were some limitations in this study. First, only eight studies meeting inclusion criteria were included into the meta-analysis. Second, it may not be appropriate to pool HRs and ORs directly without data conversion, though the subgroup analysis indicated that the overall result was not significantly affected by the different risk expressions. Third, our study failed to explore the association of different types of dementia, because most studies did not specifically report the subtypes of dementia, with only one study separately stating the subtypes of Alzheimer's disease and vascular dementia (Takeuchi et al., 2017).

In conclusion, the results from this study indicated that tooth loss may be a risk factor for dementia, and dementia risk may increase with the increase in the number of teeth los. In addition, timely prosthodontic treatment with dentures may reduce the risk of dementia related to tooth loss. Therefore, in clinical practice, tooth loss should be regarded as a marker for some systemic disorders, including dementia. Moreover, necessary intervention should be adopted for tooth loss. However, it remains to be determined whether removable dentures can be safely employed in tooth loss patients with dementia. Internal connections of chewing ability, nutrient intakes, increased inflammatory markers, and psychosocial factors, as well as clarifying the susceptibility among tooth loss patients to the various subtypes of dementia, should be further explored.

## Author Contributions

JC, W-DL, and X-TZ designed the study. C-JR and L-YX performed the data searches and collected the data, JS re-checked the data. LW and X-TZ performed the analysis. JC and LW wrote the manuscript, W-DL and X-TZ reviewed the manuscript.

### Conflict of Interest Statement

The authors declare that the research was conducted in the absence of any commercial or financial relationships that could be construed as a potential conflict of interest.

## References

[B1] Arrive E. Letenneur L. Matharan F. Laporte C. Helmer C. Barberger-Gateau P.. (2012). Oral health condition of French elderly and risk of dementia: a longitudinal cohort study. Community Dent. Oral. Epidemiol. 40, 230–238. 10.1111/j.1600-0528.2011.00650.x 22059867

[B2] Banu R. F. Veeravalli P. T. Kumar V. A. (2016). Comparative evaluation of changes in brain activity and cognitive function of edentulous patients, with dentures and two-implant supported mandibular overdenture-pilot study. Clin. Implant. Dent. Relat. Res. 18, 580–587. 10.1111/cid.12336 25825258

[B3] Batty G. D. Li Q. Huxley R. Zoungas S. Taylor B. A. Neal B.. (2013). Oral disease in relation to future risk of dementia and cognitive decline: prospective cohort study based on the Action in Diabetes and Vascular Disease: Preterax and Diamicron Modified-Release Controlled Evaluation (ADVANCE) trial. Eur. Psychiatry 28, 49–52. 10.1016/j.eurpsy.2011.07.005 21964484 PMC4170753

[B4] Busby M. Martin J. A. Matthews R. Burke F. J. Chapple I. (2014). The relationship between oral health risk and disease status and age, and the significance for general dental practice funding by capitation. Br. Dent. J. 217:E19. 10.1038/sj.bdj.2014.1006 25415037

[B5] Chen X. Shuman S. K. Hodges J. S. Gatewood L. C. Xu J. (2010). Patterns of tooth loss in older adults with and without dementia: a retrospective study based on a Minnesota cohort. J. Am. Geriatr. Soc. 58, 2300–2307. 10.1111/j.1532-5415.2010.03192.x 21143439

[B6] Cheng F. Zhang M. Wang Q. Xu H. Dong X. Gao Z.. (2018). Tooth loss and risk of cardiovascular disease and stroke: a dose-response meta analysis of prospective cohort studies. PLoS ONE 13:e0194563. 10.1371/journal.pone.0194563 29590166 PMC5874035

[B7] Dursun E. Gezen-Ak D. Hanagasi H. Bilgic B. Lohmann E. Ertan S. (2015). The interleukin 1 alpha, interleukin 1 beta, interleukin 6 and alpha-2-macroglobulin serum levels in patients with early or late onset Alzheimer's disease, mild cognitive impairment or Parkinson's disease. J. Neuroimmunol. 283, 50–57. 10.1016/j.jneuroim.2015.04.014 26004156

[B8] Egger M. Davey Smith G. Schneider M. Minder C. (1997). Bias in meta-analysis detected by a simple, graphical test. BMJ 315, 629–634. 10.1136/bmj.315.7109.629 9310563 PMC2127453

[B9] Engelborghs S. Gilles C. Ivanoiu A. Vandewoude M. (2014). Rationale and clinical data supporting nutritional intervention in Alzheimer's disease. Acta Clin. Belg. 69, 17–24. 10.1179/0001551213Z.0000000006 24635394

[B10] Greenland S. Longnecker M. P. (1992). Methods for trend estimation from summarized dose-response data, with applications to meta-analysis. Am. J. Epidemiol. 135, 1301–1309. 10.1093/oxfordjournals.aje.a116237 1626547

[B11] Jayedi A. Rashidy-Pour A. Shab-Bidar S. (2018). Vitamin D status and risk of dementia and Alzheimer's disease: a meta-analysis of dose-response. Nutr. Neurosci. 10.1080/1028415X.2018.1436639. [Epub ahead of print]. 29447107

[B12] Jimenez M. Giovannucci E. Krall Kaye E. Joshipura K. J. Dietrich T. (2014). Predicted vitamin D status and incidence of tooth loss and periodontitis. Public Health Nutr. 17, 844–852. 10.1017/S1368980013000177 23469936 PMC4911807

[B13] Kamer A. R. Craig R. G. Pirraglia E. Dasanayake A. P. Norman R. G. Boylan R. J.. (2009). TNF-alpha and antibodies to periodontal bacteria discriminate between Alzheimer's disease patients and normal subjects. J. Neuroimmunol. 216, 92–97. 10.1016/j.jneuroim.2009.08.013 19767111 PMC2783848

[B14] Kim J. M. Stewart R. Prince M. Kim S. W. Yang S. J. Shin I. S.. (2007). Dental health, nutritional status and recent-onset dementia in a Korean community population. Int. J. Geriatr. Psychiatry 22, 850–855. 10.1002/gps.1750 17266172

[B15] Kisely S. Baghaie H. Lalloo R. Siskind D. Johnson N. W. (2015). A systematic review and meta-analysis of the association between poor oral health and severe mental illness. Psychosom. Med. 77, 83–92. 10.1097/PSY.0000000000000135 25526527

[B16] Kothari M. Spin-Neto R. Nielsen J. F. (2016). Comprehensive oral-health assessment of individuals with acquired brain-injury in neuro-rehabilitation setting. Brain. Inj. 30, 1103–1108. 10.3109/02699052.2016.1167244 27260784

[B17] Koyama A. O'brien J. Weuve J. Blacker D. Metti A. L. Yaffe K. (2013). The role of peripheral inflammatory markers in dementia and Alzheimer's disease: a meta-analysis. J. Gerontol. A Biol. Sci. Med. Sci. 68, 433–440. 10.1093/gerona/gls187 22982688 PMC3693673

[B18] Lafon A. Pereira B. Dufour T. Rigouby V. Giroud M. Bejot Y.. (2014). Periodontal disease and stroke: a meta-analysis of cohort studies. Eur. J. Neurol. 21, 1155–1161, e1166–e1157. 10.1111/ene.12415 24712659

[B19] Lawton D. M. Gasquoine P. G. Weimer A. A. (2015). Age of dementia diagnosis in community dwelling bilingual and monolingual Hispanic Americans. Cortex 66, 141–145. 10.1016/j.cortex.2014.11.017 25598395 PMC4426973

[B20] Lehrer S. Green S. Rosenzweig K. E. (2015). Lack of correlation between benign brain tumors and markers of oral health. N.Y. State Dent. J. 81, 41–43. 26094363

[B21] Liesz A. Roth S. Zorn M. Sun L. Hofmann K. Veltkamp R. (2015). Acquired Immunoglobulin G deficiency in stroke patients and experimental brain ischemia. Exp. Neurol. 271, 46–52. 10.1016/j.expneurol.2015.04.021 25959599

[B22] Lin C. S. (2018). Revisiting the link between cognitive decline and masticatory dysfunction. BMC Geriatr. 18:5. 10.1186/s12877-017-0693-z 29304748 PMC5756393

[B23] Luraschi J. Korgaonkar M. S. Whittle T. Schimmel M. Muller F. Klineberg I. (2013). Neuroplasticity in the adaptation to prosthodontic treatment. J. Orofac. Pain 27, 206–216. 10.11607/jop.1097 23882453

[B24] Mayeux R. (2003). Epidemiology of neurodegeneration. Annu. Rev. Neurosci. 26, 81–104. 10.1146/annurev.neuro.26.043002.094919 12574495

[B25] Melsen W. G. Bootsma M. C. Rovers M. M. Bonten M. J. (2014). The effects of clinical and statistical heterogeneity on the predictive values of results from meta-analyses. Clin. Microbiol. Infect. 20, 123–129. 10.1111/1469-0691.12494 24320992

[B26] Minn Y. K. Suk S. H. Park H. Cheong J. S. Yang H. Lee S.. (2013). Tooth loss is associated with brain white matter change and silent infarction among adults without dementia and stroke. J. Korean. Med. Sci. 28, 929–933. 10.3346/jkms.2013.28.6.929 23772160 PMC3678012

[B27] Moher D. Liberati A. Tetzlaff J. Altman D. G. (2009). Preferred reporting items for systematic reviews and meta-analyses: the PRISMA statement. PLoS Med. 6:e1000097 10.1371/journal.pmed.1000097 19621072 PMC2707599

[B28] Nascimento G. G. Leite F. R. Conceicao D. A. Ferrua C. P. Singh A. Demarco F. F. (2016). Is there a relationship between obesity and tooth loss and edentulism? A systematic review and meta-analysis. Obes. Rev. 17, 587–598. 10.1111/obr.12418 27125768

[B29] Oh B. Han D. H. Han K. T. Liu X. Ukken J. Chang C.. (2018). Association between residual teeth number in later life and incidence of dementia: a systematic review and meta-analysis. BMC Geriatr. 18:48. 10.1186/s12877-018-0729-z 29454307 PMC5816354

[B30] Orsini N. Li R. Wolk A. Khudyakov P. Spiegelman D. (2012). Meta-analysis for linear and nonlinear dose-response relations: examples, an evaluation of approximations, and software. Am. J. Epidemiol. 175, 66–73. 10.1093/aje/kwr265 22135359 PMC3244608

[B31] Paganini-Hill A. White S. C. Atchison K. A. (2012). Dentition, dental health habits, and dementia: the Leisure World Cohort Study. J. Am. Geriatr. Soc. 60, 1556–1563. 10.1111/j.1532-5415.2012.04064.x 22860988

[B32] Perera R. Ekanayake L. (2012). Relationship between nutritional status and tooth loss in an older population from Sri Lanka. Gerodontology 29, e566–570. 10.1111/j.1741-2358.2011.00518.x 21771051

[B33] Pillai R. S. Iyer K. Spin-Neto R. Kothari S. F. Nielsen J. F. Kothari M. (2018). Oral health and brain injury: causal or casual relation? Cerebrovasc. Dis. Extra. 8, 1–15. 10.1159/000484989 29402871 PMC5836263

[B34] Savikko N. Saarela R. K. Soini H. Muurinen S. Suominen M. H. Pitkala K. H. (2013). Chewing ability and dementia. J. Am. Geriatr. Soc. 61, 849–851. 10.1111/jgs.12233 23672568

[B35] Shen T. Lv J. Wang L. Wang W. Zhang D. (2016). Association between tooth loss and dementia among older people: a meta-analysis. Int. J. Geriatr. Psychiatry 31, 953–955. 10.1002/gps.4396 26644219

[B36] Shi J. Leng W. Zhao L. Deng C. Xu C. Wang J.. (2018). Tooth loss and cancer risk: a dose-response meta analysis of prospective cohort studies. Oncotarget 9, 15090–15100. 10.18632/oncotarget.23850 29599929 PMC5871100

[B37] Shimazaki Y. Soh I. Saito T. Yamashita Y. Koga T. Miyazaki H.. (2001). Influence of dentition status on physical disability, mental impairment, and mortality in institutionalized elderly people. J. Dent. Res. 80, 340–345. 10.1177/00220345010800010801 11269726

[B38] Singhrao S. K. Harding A. Simmons T. Robinson S. Kesavalu L. Crean S. (2014). Oral inflammation, tooth loss, risk factors, and association with progression of Alzheimer's disease. J. Alzheimers Dis. 42, 723–737. 10.3233/JAD-140387 24946875

[B39] Song F. Gilbody S. (1998). Bias in meta-analysis detected by a simple, graphical test. Increase in studies of publication bias coincided with increasing use of meta-analysis. BMJ 316:471. 9492690 PMC2665616

[B40] Stein P. S. Desrosiers M. Donegan S. J. Yepes J. F. Kryscio R. J. (2007). Tooth loss, dementia and neuropathology in the Nun study. J. Am. Dent. Assoc. 138, 1314–1322. 10.14219/jada.archive.2007.0046 17908844

[B41] Stewart R. Stenman U. Hakeberg M. Hagglin C. Gustafson D. Skoog I. (2015). Associations between oral health and risk of dementia in a 37-year follow-up study: the prospective population study of women in Gothenburg. J. Am. Geriatr. Soc. 63, 100–105. 10.1111/jgs.13194 25597561

[B42] Takeuchi K. Ohara T. Furuta M. Takeshita T. Shibata Y. Hata J.. (2017). Tooth loss and risk of dementia in the community: the hisayama study. J. Am. Geriatr. Soc. 65, e95–e100. 10.1111/jgs.14791 28272750

[B43] Tang X. Pan Y. Zhao Y. (2013). Vitamin D inhibits the expression of interleukin-8 in human periodontal ligament cells stimulated with Porphyromonas gingivalis. Arch. Oral. Biol. 58, 397–407. 10.1016/j.archoralbio.2012.09.010 23083515

[B44] Teixeira F. B. Pereira Fernandes Lde M. Noronha P. A. Dos Santos M. A. Gomes-Leal W. Ferraz Maia Cdo S.. (2014). Masticatory deficiency as a risk factor for cognitive dysfunction. Int. J. Med. Sci. 11, 209–214. 10.7150/ijms.6801 24465167 PMC3894406

[B45] Tonsekar P. P. Jiang S. S. Yue G. (2017). Periodontal disease, tooth loss and dementia: is there a link? A systematic review. Gerodontology 34, 151–163. 10.1111/ger.12261 28168759

[B46] Yamamoto T. Kondo K. Hirai H. Nakade M. Aida J. Hirata Y. (2012). Association between self-reported dental health status and onset of dementia: a 4-year prospective cohort study of older Japanese adults from the Aichi Gerontological Evaluation Study (AGES) Project. Psychosom. Med. 74, 241–248. 10.1097/PSY.0b013e318246dffb 22408130

[B47] Zeng X. T. Luo W. Huang W. Wang Q. Guo Y. Leng W. D. (2013). Tooth loss and head and neck cancer: a meta-analysis of observational studies. PLoS ONE 8:e79074. 10.1371/journal.pone.0079074 24260154 PMC3829962

